# Identifying Individuals at High Risk of Psychosis: Predictive Utility of Support Vector Machine using Structural and Functional MRI Data

**DOI:** 10.3389/fpsyt.2016.00052

**Published:** 2016-04-08

**Authors:** Isabel Valli, Andre F. Marquand, Andrea Mechelli, Marie Raffin, Paul Allen, Marc L. Seal, Philip McGuire

**Affiliations:** ^1^Department of Psychosis Studies, Institute of Psychiatry, King’s College London, London, UK; ^2^Donders Institute for Brain, Cognition and Behaviour, Radboud University, Nijmegen, Netherlands; ^3^Centre for Neuroimaging Sciences, King’s College London, London, UK; ^4^Service de Psychiatrie de l’enfant et de l’adolescent, CHU Pitié-Salpêtrière, Paris, France; ^5^Developmental Imaging Research Group, Murdoch Children Research Institute, University of Melbourne, Melbourne, VIC, Australia

**Keywords:** psychosis, risk, support vector machine, MRI and fMRI, at-risk mental state, schizophrenia, verbal learning, memory

## Abstract

The identification of individuals at high risk of developing psychosis is entirely based on clinical assessment, associated with limited predictive potential. There is, therefore, increasing interest in the development of biological markers that could be used in clinical practice for this purpose. We studied 25 individuals with an at-risk mental state for psychosis and 25 healthy controls using structural MRI, and functional MRI in conjunction with a verbal memory task. Data were analyzed using a standard univariate analysis, and with support vector machine (SVM), a multivariate pattern recognition technique that enables statistical inferences to be made at the level of the individual, yielding results with high translational potential. The application of SVM to structural MRI data permitted the identification of individuals at high risk of psychosis with a sensitivity of 68% and a specificity of 76%, resulting in an accuracy of 72% (*p* < 0.001). Univariate volumetric between-group differences did not reach statistical significance. By contrast, the univariate fMRI analysis identified between-group differences (*p* < 0.05 corrected), while the application of SVM to the same data did not. Since SVM is well suited at identifying the *pattern of abnormality* that distinguishes two groups, whereas univariate methods are more likely to identify regions that *individually* are most different between two groups, our results suggest the presence of focal functional abnormalities in the context of a diffuse pattern of structural abnormalities in individuals at high clinical risk of psychosis.

## Introduction

At present, it is possible to identify individuals at a greatly increased risk of developing psychosis on the basis of clinical features that may include attenuated psychotic symptoms (1), schizotypal personality traits (2), a positive family history of psychosis, and a marked decline in overall function (3). Individuals presenting with these features are defined as having an at-risk mental state (ARMS) (1), which is associated with a risk of subsequent transition to psychosis of 18% after 6 months of follow-up, 22% after 1 year, 29% after 2 years, and 36% after 3 years (4). However, there is increasing evidence that psychotic experiences are quite common among adolescents and young adults in the general population (5), and the particular clinical criteria that should be used are still being debated (6). This has led to interest in neurobiological markers of psychosis risk.

Structural magnetic resonance imaging (sMRI) studies have provided robust evidence for structural brain abnormalities in high-risk populations, with the most pronounced gray matter (GM) differences relative to healthy controls observed in the prefrontal, cingulate, lateral, and medial temporal cortices (7, 8). These regions are critical for episodic memory performance, which is reported to be impaired in the ARMS (9). Verbal episodic memory deficits were found to be associated with reductions in GM volume (GMV), particularly in the medial temporal cortex, not only in the ARMS (10) but also in non-psychotic relatives of individuals with schizophrenia (11). A small number of studies have investigated the functional correlates of episodic memory dysfunction in the ARMS (9), including work from our group on a sample partially overlapping with the present one, which identified activation abnormalities in brain regions, including prefrontal and medial temporal cortices (12, 13). These studies were conducted with a univariate analytical approach. Multivariate analysis methods, such as support vector machine (SVM)(14, 15), offer the advantage of enabling statistical inferences to be made at the level of the individual and, therefore, yield results with high translational potential in clinical practice. In particular, SVM is a technique for classifying individual observations into distinct groups or classes, based on the detection of regularities in high-dimensional data (16). The use of multivariate analysis approaches has previously demonstrated the potential of structural MRI data for the discrimination of patients with schizophrenia from healthy controls, with 81 and 86% diagnostic accuracies observed for patients with chronic schizophrenia (15) and first-episode patients (17), respectively. Considerable interest lays in the potential of these approaches to identify individuals at risk of the disorder and to identify which individuals will develop psychosis among those that show a vulnerability to it. A recent study addressing this issue found that the structural neuroanatomy of high-risk individuals provided information that permitted their distinction from controls with an 86% accuracy, irrespective of clinical outcome, and further indicated that structural abnormalities were most pronounced in the individuals who went on to develop psychosis (18). The first study using a pattern classification approach for the analysis of neurocognitive parameters in high-risk individuals obtained a 94.2% accuracy in distinguishing vulnerable individuals from healthy controls, with discriminatory patterns involving mainly verbal learning and memory domains (19). A further analysis aiming to discriminate individuals that subsequently developed psychosis from the rest of the participants resulted in 90.8% accuracy, with transition to psychosis mainly predicted by executive and verbal learning impairments (19). However to date no study has investigated the potential of the functional correlates of verbal learning for identifying individuals at high-risk for psychosis using a multivariate approach. The aim of the present investigation was, therefore, to use a standard univariate analysis and a SVM classifier to examine structural imaging data and the functional correlates of a verbal memory task. We hypothesized that SVM applied to structural and functional imaging data, respectively, would permit the identification of individuals with an ARMS with statistically significant accuracies.

## Materials and Methods

The protocol of the study was in compliance with the Code of Ethics of the World Medical Association, it was approved by the research ethics committee of the Institute of Psychiatry, King’s College London, and all participants gave written informed consent to participate after a complete description of the study.

### Participants

Twenty-five individuals meeting criteria for an ARMS were recruited from Outreach and Support in South London (20), a clinical service for people at risk of developing psychosis. The diagnosis was based on the PACE criteria (21), as assessed by two expert clinicians using the Comprehensive Assessment for At-Risk Mental States (CAARMS) (22) and confirmed at a consensus clinical meeting. All subjects were antipsychotic naïve at the time of scanning, and six were receiving antidepressant medication. Twenty-five control subjects were recruited over the same period from the same sociodemographic area. Subjects were aged 18–30 years and were all native speakers of English. Participants were excluded if their IQ was below 70, if they had a history of a neurological disorder, a history of severe head injury, or if they met DSM-IV criteria for an alcohol or drug dependence disorder. An additional exclusion criterion for control subjects was a family history of psychosis. The study includes all participants reported in a previous manuscript (9) that investigated a subsample of the current ARMS and control groups.

All participants, with the exception of one for each group, were right handed as evaluated using the Lateral Preferences Inventory (23).

### Clinical Measures

Current symptoms were assessed in all participants at the time of scanning using the Positive and Negative Syndrome Scale (PANSS) (24). Premorbid IQ was estimated with the Wide Range Achievement Test-Revised (WRAT-R) (25).

### Statistical Analyses

Statistical analyses were conducted using SPSS version 16.0 (SPSS inc. Chicago, IL, USA). Student’s *t*-test was used to compare ARMS and control participants in terms of demographic and clinical variables.

### Image Acquisition

Images were acquired using a 1.5-T GE NV/I Signa LX Horyzon system (General Electric, Milwaukee, WI, USA) at the Centre for Neuroimaging Sciences, King’s College London.

#### Structural Images

T1-weighted inversion recovery spoiled gradient structural images were acquired with the following acquisition parameters: TE = 5200 ms, TR = 15900 ms, flip angle = 20°, field of view = 220 mm × 176 mm, slice thickness = 1.5 mm, number of slices = 124, image matrix = 256 × 256 × 124.

#### Functional Images

T2-weighted images were acquired using gradient-echo echoplanar magnetic resonance imaging (EPI) during the Encoding and the Recognition condition of an episodic verbal memory paradigm previously described (12). The data reported here refer to the encoding condition, in which 160 words were visually presented in blocks of 10 words each, back projected with an LCD projector on to a screen viewed through a prism positioned over the head coil. Participants were asked to read words aloud and try to remember them. Stimuli were presented with stimulus onset asynchrony of 4 s. Between each encoding block, there was a word repetition condition in which subjects were required to repeatedly view and say the word “wait” in blocks of four presentations each. Participants were aware that the encoding task would be followed by a test of recognition of the presented material. During the encoding condition, 228 image volumes were acquired in a single functional run using a compressed acquisition sequence (TR = 4000 ms, silent period 2500 ms) to allow verbal articulation of the stimuli in the absence of acoustic scanner noise and to minimize motion artifacts related to overt articulation. Images were acquired in 16 non-contiguous axial planes parallel to the intercommissural plane with the following parameters: TE = 40 ms, slice thickness = 7 mm, slice skip = 0.7 mm, in plane resolution 3 mm × 3 mm.

### Univariate Analysis

#### Structural Imaging Analysis

Group-related differences in GMV were analyzed using voxel-based morphometry (VBM), implemented in SPM8 software^1^ running under Matlab 7.4 (MathWorks, Natick, MA, USA). Prior to the VBM analysis T1-weighted volumetric images were preprocessed using the Diffeomorphic Anatomical Registration Through Exponentiated Lie algebra (DARTEL) (26) SPM8 toolbox, aimed at maximizing analysis accuracy and sensitivity through the creation of a sample-specific template that is used for the segmentation of each individual image (27). After the image origin was set manually to the anterior commissure, T1-weighted images were segmented into GM, white matter (WM), and cerebrospinal fluid (CSF). GM segments were iteratively registered by non-linear warping to a template generated using DARTEL to obtain a high-dimensional normalization (26). A homogeneity check across the sample was followed by smoothing with an 8 mm full width at half maximum (FWHM) Gaussian kernel. The normalization protocol included a “modulatory step” in order to preserve information about the absolute GM values (28). After preprocessing, normalized modulated smoothed data were used for the statistical analysis. This was performed using SPM8 to compare GM images from ARMS participants and controls with a two-sample *t*-test. Age, gender, and medication were modeled in the analysis to reduce the potential impact of these variables on the findings. In order to identify specific changes not confounded by global volumetric differences, the proportional scaling option was used. Statistical inferences for the standard univariate analysis were made whole brain voxel-wise at *p* < 0.05 family wise error (FWE).

#### Functional Imaging Analysis

Functional images were realigned to the first volume in the series to correct for movement during acquisition, transformed into a standard space (SPM EPI template) and smoothed using a 6-mm isotropic Gaussian filter using SPM5 (Wellcome Department of Imaging Neuroscience, London, UK, see text footnote 1) running in matlab 7.4 (MathWorks, Natick, MA, USA). A standard random effects statistical analysis of regional responses was performed to identify regional activations in each subject independently. To remove low-frequency drifts, the data were high-pass filtered using a set of discrete cosine basis functions with a cut off period of 128 s.

Each stimulus was modeled independently by convolving the onset times with the hemodynamic response function (HRF). First, the parameter estimates for encoding and repetition were calculated using the general linear model in each individual subject. Second a group analysis was performed using a 2 × 2 factorial model of variance (ANOVA), with group (ARMS, controls) and condition (encoding, repetition) as factors. Age, gender, and medication were modeled as covariates to minimize the impact of these potentially confounding variables on the results. The whole brain voxel-wise threshold was set at *p* < 0.05 FWE corrected.

Regression coefficients (“beta values”), which provide information about the fit of the regressor to the data at each voxel, were obtained for each subject, masked to include only intracerebral tissue, then used for the multivariate analysis of the Encoding condition.

### Multivariate Analysis

A linear SVM was used to classify ARMS participants and controls on the basis of their brain structure. A separate analysis was performed on the basis of their brain activation during the Encoding phase of a verbal episodic memory task. The SVM classifier was implemented using the PROBID software^2^ (Institute of Psychiatry, London, UK) running in matlab 7.4. (MatWorks, Natick, MA, USA).

#### Classification and Support Vector Machine

Support vector machine is a supervised multivariate classification method where “supervised” refers to a training step in which the algorithm learns the differences between pre-specified groups to be classified (29). SVM treats each image as a point in a high-dimensional space, where the number of dimensions equals the number of intracerebral voxels. Participants were allocated to one of two classes (ARMS or controls) and a linear classification function was learnt from the imaging data in order to discriminate between the two groups. To linearly classify the data, a decision boundary or hyperplane (a generalization of a plane of *n* − 1 dimensions that splits an *n*-dimensional space) must be defined in order to separate the data based on class membership. However, for a linearly separable problem, there are infinitely many hyperlanes that correctly classify the data. The SVM algorithm (30) finds the optimal one in the sense that it is characterized by the largest margin between classes. The margin is defined as the distance of the closest training data-points to the hyperplane. These points are the most difficult to classify and are called support vectors. The hyperplane is defined by a weight vector, which is a linear combination of the support vectors and specifies both a direction and an offset that together define the maximum margin classifier. The SVM regularization parameter (conventionally denoted “C”) was fixed to its default value (1), following common practice in neuroimaging studies.

#### Discrimination Maps

The weight vector is normal to the hyperplane and can be conceptualized as a spatial representation of the decision boundary. It has the same dimension as the training data (in this case voxel space) and can, therefore, provide a map of the most discriminatory regions (discrimination map). If the two groups are attributed labels of +1 and −1, higher positive values indicate regions making a positive contribution toward identifying the first group relative to the second and vice versa, indicating which regions are most important for defining class membership for the first and the second group, respectively [for a description see Ref. (31)]. In the present study, positive values were associated with the ARMS group and coded in red/yellow color scale, while the control group was associated with a negative weight and coded in blue/cyan color scale. The multivariate nature of the classifier provides a spatial pattern of regions that discriminate between the two groups.

#### Classifier Performance

The performance of the classifier was assessed using a leave-one-out cross-validation procedure (16). This approach consists of training the classifier with all participants except from one pair and subsequently testing the group membership assigned to the excluded subjects. This test was repeated 25 times, each time excluding a different pair of participants, one from the ARMS group and one from the control group. This procedure permitted the measurement of the accuracy of the classifier, defined as the proportion of correctly classified participants. It also permitted the quantification of the sensitivity and specificity of the classifier, defined as follows:

– Sensitivity = TP/(TP+FN)
– Specificity = TN/(TN+FP)

TP refers to true positives, which is the number of individuals from the ARMS group correctly classified. TN, or true negatives, is the number of individuals from the control group correctly classified. FP, or false positives, refers to the number of control participants misclassified as belonging to the ARMS group, while FN, or false negatives, is the number of ARMS participants misclassified as belonging to the control group.

#### Permutation Testing

The significance of the SVM classification was assessed at whole-brain level using a non-parametric permutation test. This evaluates the probability of obtaining by chance sensitivity and specificity values higher than those obtained during the leave-one-out cross-validation procedure. This method randomly assigns participants to one of the classes before training the SVM. In the present study, 1000 permutations were conducted and the null hypothesis of the observed classification being observed by chance was rejected for *p* = 0.05. The discrimination maps show the most important regions contributing to an overall accuracy significant at the *p* < 0.05 level.

## Results

### Demographic and Clinical Variables

The two groups were matched in terms of age and estimated premorbid IQ. There was a larger proportion of male participants in the ARMS group (72%) than in the control group (56%), though not statistically significant.

As expected, the two groups differed in terms of symptom severity as assessed using the PANSS total score and each of the subscales (Table 1).

**Table 1:** **Demographic and clinical variables by group**.

	Group		Group comparison
	ARMS (***N*** = 25)	Controls (***N*** = 25)	
Age (years)	23.84 (5.52)	25.12 (3.21)	*t* = −1.003, *p* = 0.322
*N*, Male/Female	18/7	14/11	χ^2^ = 1.389, df = 1, *p* = 0.239
Premorbid IQ	101.48 (13.20)	104.12 (8.80)	*t* = −0.832, *p* = 0.410
PANSS	46.40 (9.51)	30.44 (0.96)	*t* = 8.35, *p* < 0.001
PANSS positive	12.48 (3.19)	7.2 (0.50)	*t* = 8.18, *p* < 0.001
PANSS negative	10.16 (4.06)	7.00 (0.00)	*t* = 3.89, *p* < 0.001
PANSS general	23.80 (5.45)	16.24 (0.66)	*t* = 6.88, *p* < 0.001

*Data reflect mean (and SD). Df (degrees of freedom) = 48; *N* = number; PANSS, Positive and Negative Syndrome Scale*.

### Imaging Results

#### Univariate Structural Analysis

When VBM was used, volumetric between-group differences were observed only at uncorrected level but none reached a trend for significance with correction for multiple comparisons (*p* < 0.05, FWE corrected).

#### Univariate Functional Analysis

**Figure 1:**
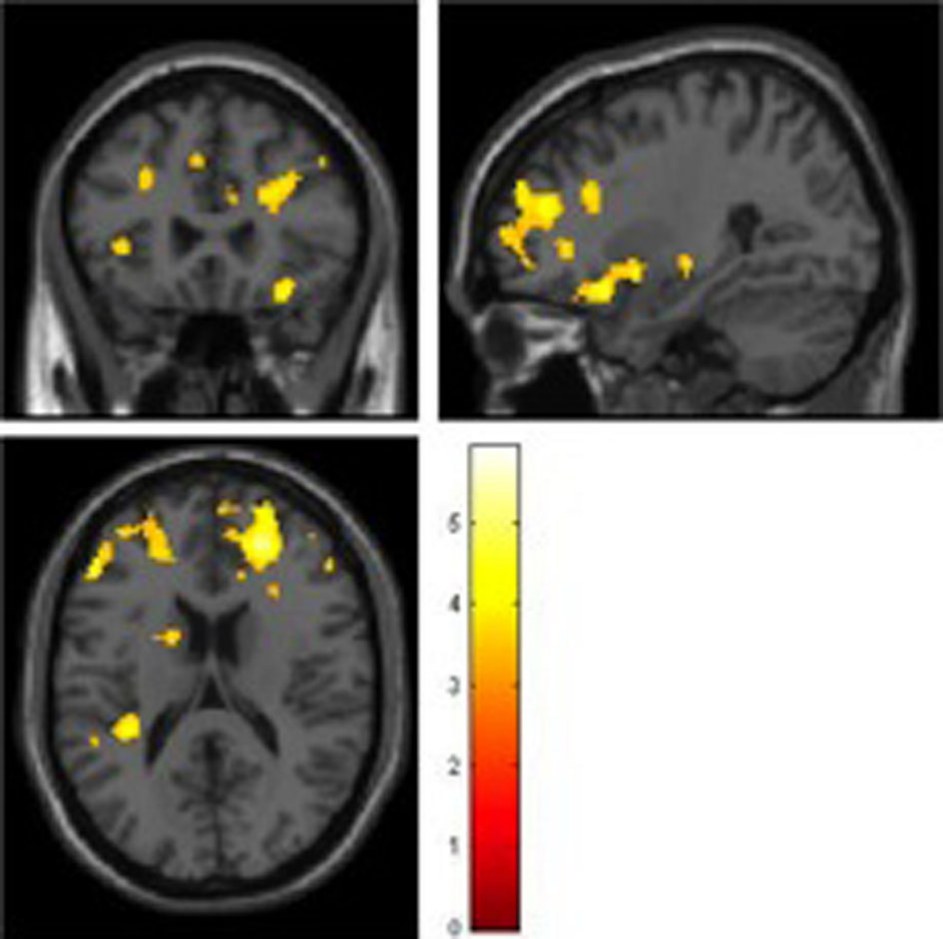
**Main effect of Encoding (FWE corrected)**.

During the encoding condition, controls showed greater activation than ARMS subjects in the left middle frontal and precentral gyri, supramarginal gyrus, and insula as well as the right medial frontal gyrus (Table 2, Figure 1).

**Table 2:** **Main effect of Encoding**.

Brain region	*x*	*y*	*z*	*z*-score
				
L insula	−32	−24	4	5.46
L precentral gyrus	−32	6	42	5.34
R medial frontal gyrus	22	44	20	5.11
L middle frontal gyrus	−52	34	20	4.74
L supramarginal gyrus	−46	−52	24	4.56

#### Multivariate Structural Analysis

The structural neuroanatomy of individuals at risk of psychosis permitted their discrimination from healthy controls at a statistically significant level (*p* < 0.001) with 68% sensitivity and 76% specificity, resulting in 72% accuracy.

**Figure 2:**
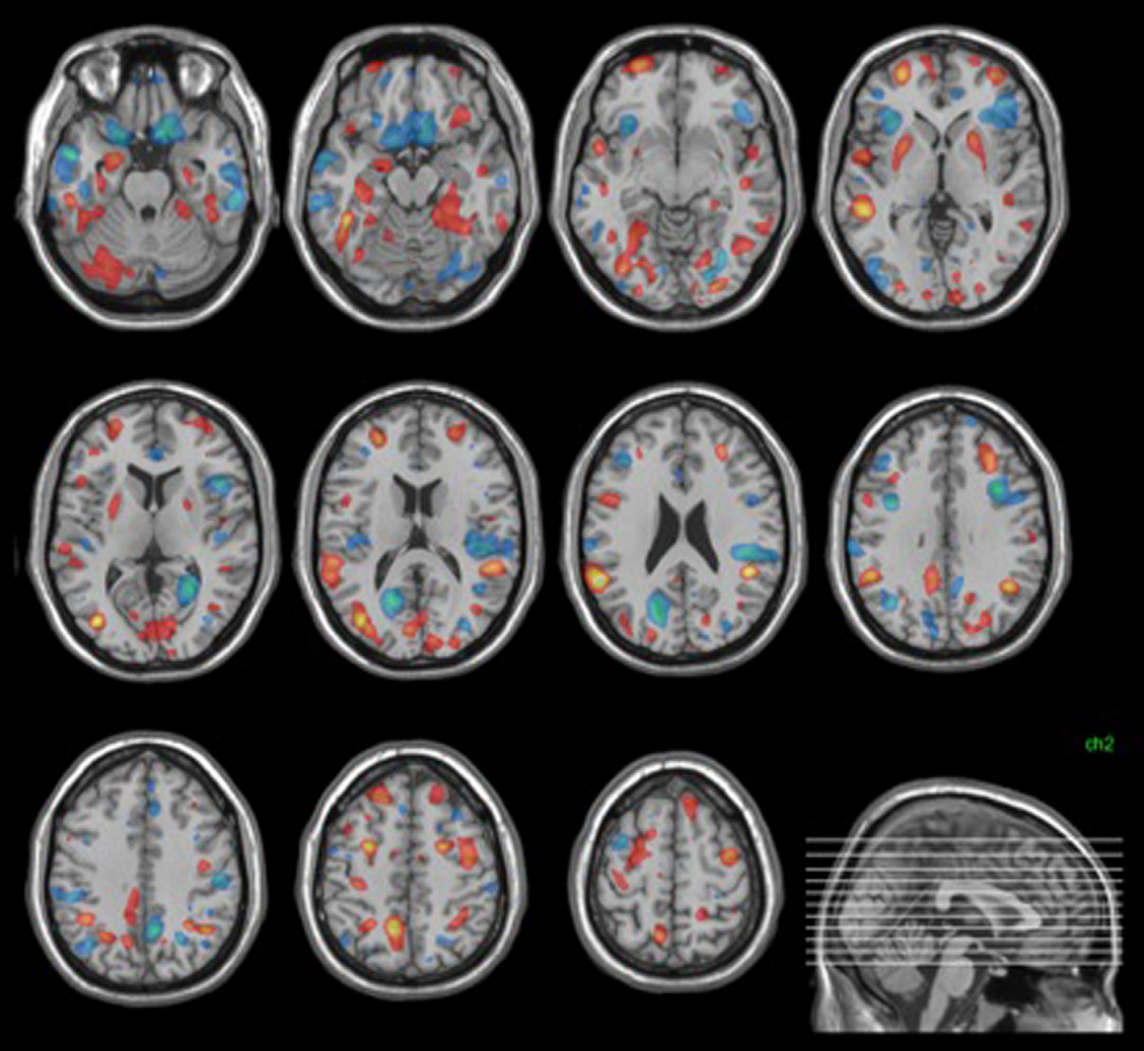
**Structural discrimination map**. Areas shown in red were those most distinctive of ARMS group membership. Those in blue were most distinctive of control group membership. Images were thresholded to show the top 30% of voxel weight vector values (positive and negative).

The neuroanatomical pattern distinctive of the ARMS group (i.e., having high magnitude positive weights) showed high weights bilaterally in the hippocampus, parahippocampal gyrus, putamen, superior and middle frontal gyri, middle temporal gyrus, fusiform gyrus, and inferior parietal lobule. In addition, lateralized findings distinctive of ARMS group membership included the left inferior temporal gyrus, right superior temporal gyrus, left precuneus, and left cerebellum (Figure 2).

The pattern distinctive of the control group (i.e., having high magnitude negative weights) showed high weights bilaterally in the medial and inferior frontal gyri, the inferior and middle temporal gyri, the insula, the cuneus, the cerebellum, anterior, and posterior cingulate. Lateralized findings distinctive of control group membership included the left superior and middle frontal gyri, the left middle occipital gyrus and right precuneus (Figure 2).

#### Multivariate Functional Analysis

Based on the functional imaging data, no significant discrimination of ARMS subjects relative to controls was obtained, with between-group discrimination no greater than chance (*p* = 0.34), sensitivity of 48%, specificity of 60%, and overall accuracy of 54% when the leave-one-out cross-validation procedure was employed.

## Discussion

Identification of psychosis prone individuals still uses a method available 100 years ago – a clinical interview. In the rest of medicine, investigations often involve the use of biological tests, which can help to identify conditions of risk, and allow earlier detection of the disorder. In the absence of diagnostic biomarkers for psychosis, methods that permit the distinction of vulnerable individuals from healthy controls have important implications for the early detection and diagnosis of the disorder.

Structural and functional MRI data were used to assess their potential to reliably distinguish control and ARMS individuals using multivariate analysis techniques.

The results indicated that the multivariate approach enabled the identification of structural differences that distinguished the two groups. By contrast, the univariate analysis of the functional MRI data identified significant group differences, while the SVM method did not. In the present study, the sample size may have limited the power to identify group differences, but their detection with one analytical method rather than the other may alternatively reflect a different distribution of structural and functional abnormalities. Univariate analytical approaches consider each voxel independently and are well suited to detecting effects that are localized and robust; by contrast, multivariate methods take into account between-voxel correlations and are ideal for detecting subtle and spatially distributed patterns of abnormality (32). If SVM is well suited at finding the *set of areas* that jointly distinguish two groups, whereas univariate methods are more likely to identify regions that *individually* are most different between two groups, our results suggest the presence of focal functional abnormalities in the context of a diffuse pattern of structural abnormalities.

Based on previous SVM findings, both in schizophrenia (15, 17) and in the ARMS (32), it was predicted that structural MRI data would allow a robust between-group discrimination. In the present sample, using a leave-one-out procedure, the whole-brain structural correlates of the ARMS identified individuals belonging to this group relative to healthy controls with 72% accuracy. This is lower relative to accuracy levels previously reported for schizophrenia (15, 17), and in a previous MRI study in the ARMS (18). Nevertheless, the classification was highly significant under permutation (*p* < 0.001). The results, therefore, replicate previous findings, albeit with a relatively lower accuracy, and suggest that the high risk of psychosis seen in people with an ARMS is associated with significant alterations in brain anatomy.

Some of the regions identified as most important for discriminating between ARMS and controls feature within circuits connecting the medial temporal region to the lateral temporal and prefrontal cortices (33), networks that normally play an important role in episodic memory (34). However, the SVM analysis of the functional MRI data acquired during an episodic memory task failed to reliably distinguish the ARMS participants from healthy controls. These data, thus, suggest that activation during the encoding phase of an episodic memory task may not have potential for the identification of group membership in this context. Nevertheless, we cannot exclude the possibility that a significant difference might have emerged had the sample size been larger. A previous investigation, which used fMRI in conjunction with an *n*-back working memory task to discriminate between ARMS individuals and healthy controls (35), reported a statistically significant accuracy of 76.2%. However, we note that this investigation was also carried out in a small sample of 19 ARMS and 19 healthy controls. In order to address the apparent inconsistency between the results of this previous investigation and those of our study, replication with a larger sample size and multiple memory tasks (e.g., working vs. episodic memory) would be required, especially considering the clinical heterogeneity of the population under investigation.

When structural imaging data are considered using machine learning methods, the findings cannot be interpreted simply in terms of greater or lower GMV in one group relative to the other. The set of areas identified in each group represent the brain regions that are the most important for predicting membership of each group. Regions in the predictive pattern can be assigned high weight vector scores either because of a large difference in GMV, or because the region adds predictive value by virtue of its correlation with other brain regions [e.g., to cancel out noise (36)]. In previous studies of schizophrenia, the predictive pattern from the multivariate analysis mostly implicated regions similar to those where volumetric reductions had been identified using univariate analyses (15, 37), with differences mainly in frontal, temporal, parietal, and cingulate cortices, the medial temporal lobe and the thalamus (38, 39). Similarly, the recent application of machine learning techniques to MRI data in the ARMS characterized the high-risk population by a distributed pattern comprising frontal, temporal, limbic, and cerebellar regions (18, 32). The present SVM results derived from structural data were consistent with those from previous studies, with the classification pattern containing clusters in the prefrontal and temporal cortices, and a large bilateral cluster, including the hippocampus and parahippocampal gyrus. These are areas where volumetric abnormalities have been indentified using univariate analyses in ARMS and genetic high-risk individuals relative to controls (40–42). While data from univariate MRI studies indicate that vulnerability to psychosis is associated with GM abnormalities regardless of clinical outcome (7, 41), there is also evidence that later transition to psychosis is associated with more marked abnormalities at baseline (7, 41) and with progressive volumetric reductions between baseline and the onset of psychosis (40, 43). These findings are not mutually exclusive: it is likely that the former are correlates of increased risk (independent of subsequent clinical outcome), while the latter are specific correlates of later illness. The first studies to address the issue of abnormalities specific to those subjects who will develop psychosis have provided promising results, with machine learning classifiers appearing able to distinguish subjects that would subsequently develop psychosis from those who would not, based on structural abnormalities present before psychosis onset (18). However, the clinical follow-up of the ARMS participants is still ongoing and no direct comparison was performed based on outcome; therefore, no inferences can be made relative to abnormalities specific to later transition to psychosis.

The present study had further limitations. First, the sample sizes were relatively small, which may have limited its power to detect true group differences. As discussed above, this means that the negative findings in the structural and functional analyses must be interpreted with caution, and future studies using larger samples are needed to address this issue as well as that of specificity relative to other psychiatric disorders. There was a higher proportion of male participants in the ARMS than that of the control group. The gender difference did not reach significance; however, it represents a possible confounder because of the sexual dimorphism of brain structure and development (44, 45) and the gender differences reported in brain morphology in schizophrenia (46). Finally, even though all the ARMS participants were antipsychotic naïve, six of them had been exposed to antidepressant medication. It is, therefore, not possible to rule out whether this variable may have contributed to the differences observed (47).

In conclusion, we found that a multivariate analysis of neuroanatomical images enabled the identification of individuals at high risk of psychosis with statistically significant accuracy. By contrast, the functional correlates of episodic memory did not show classification potential in this clinical population. Mass-univariate analyses are optimal for identifying focal group differences, and are more sensitive than multivariate methods if the effects are localized to particular brain regions. Multivariate methods, on the other hand, are sensitive to spatially distributed patterns of activity, and are more sensitive if the differential effects are distributed across widespread brain regions. The two analyses are, therefore, complementary and address different questions when used in combination. These results expand the current understanding of structural and functional brain abnormalities in individuals at high risk of psychosis. Future work could examine possible strategies to improve the diagnostic and prognostic classification of this clinical population, for example, through the integration of multiple modalities within a multivariate machine learning framework.

## Author Contributions

All persons designated as authors have participated in the work and have reviewed the manuscript.

## Conflict of Interest Statement

The authors declare that the research was conducted in the absence of any commercial or financial relationships that could be construed as a potential conflict of interest. The reviewer KB and handling Editor declared their shared affiliation, and the handling Editor states that the process nevertheless met the standards of a fair and objective review.

## Footnotes

^1^http://www.fil.ion.ucl.ac.uk/spm

^2^www.brainmap.co.uk/probid.htm
